# Extravillous trophoblast cells-derived exosomes promote vascular smooth muscle cell migration

**DOI:** 10.3389/fphar.2014.00175

**Published:** 2014-08-11

**Authors:** Carlos Salomon, Sarah Yee, Katherin Scholz-Romero, Miharu Kobayashi, Kanchan Vaswani, David Kvaskoff, Sebastian E. Illanes, Murray D. Mitchell, Gregory E. Rice

**Affiliations:** ^1^Centre for Clinical Diagnostics, Royal Brisbane and Women's Hospital, University of Queensland Centre for Clinical ResearchBrisbane, QLD, Australia; ^2^Department of Obstetric and Gynaecology, Faculty of Medicine, Universidad de los AndesSantiago, Chile

**Keywords:** exosomes, cell migration, placenta, pregnancy, proteomics

## Abstract

**Background:** Vascular smooth muscle cells (VSMCs) migration is a critical process during human uterine spiral artery (SpA) remodeling and a successful pregnancy. Extravillous trophoblast cells (EVT) interact with VSMC and enhance their migration, however, the mechanisms by which EVT remodel SpA remain to be fully elucidated. We hypothesize that exosomes released from EVT promote VSMC migration.

**Methods:** JEG-3 and HTR-8/SVneo cell lines were used as models for EVT. Cells were cultured at 37°C and humidified under an atmosphere of 5% CO_2_-balanced N_2_ to obtain 8% O_2_. Cell-conditioned media were collected, and exosomes (exo-JEG-3 and exo- HTR-8/SVneo) isolated by differential and buoyant density centrifugation. The effects of exo-EVT on VSMC migration were established using a real-time, live-cell imaging system (Incucyte™). Exosomal proteins where identified by mass spectrometry and submitted to bioinformatic pathway analysis (Ingenuity software).

**Results:** HTR-8/SVneo cells were significantly more (~30%) invasive than JEG-3 cells. HTR-8/SVneo cells released 2.6-fold more exosomes (6.39 × 10^8^ ± 2.5 × 10^8^ particles/10^6^ cells) compared to JEG-3 (2.86 × 10^8^ ± 0.78 × 10^8^ particles/10^6^ cells). VSMC migration was significantly increased in the presence of exo-JEG-3 and exo-HTR-8/SVneo compared to control (−exosomes) (21.83 ± 0.49 h and 15.57 ± 0.32, respectively, vs. control 25.09 ± 0.58 h, *p* < 0.05). Sonication completely abolished the effect of exosomes on VSMC migration. Finally, mass spectrometry analysis identified unique exosomal proteins for each EVT cell line-derived exosomes.

**Conclusion:** The data obtained in this study are consistent with the hypothesis that the release, content, and bioactivity of exosomes derived from EVT-like cell lines is cell origin-dependent and differentially regulates VSMC migration. Thus, an EVT exosomal signaling pathway may contribute to SpA remodeling by promoting the migration of VSMC out of the vessel walls.

## Introduction

Remodeling of the uterine spiral arteries (SpA) into low resistance, high capacity vessel begins as extravillous trophoblasts (EVT) invade the decidua during first trimester and is essential for successful pregnancy (Kam et al., 1999). When EVT “plugs” are lost during early second trimester, maternal blood flows through the modified vessels to deliver nutrients and oxygen to support fetal growth and development (Pijnenborg et al., 2006a). EVT continue to invade into the myometrium and remodel the SpA until mid-second trimester (Hamilton and Boyd, 1970; Pijnenborg et al., 1983; Blackburn et al., 2003; American Diabetes Association, 2012). The initial steps of uterine spiral artery remodeling consists of vessel dilatation, vascular smooth muscle cells (VSMC) separation, endothelial cell swelling, EVT infiltration, and fibrinoid deposition (Pijnenborg et al., 2006b). VSMC migrate or undergo apoptosis and are replaced by fibriniod material, in which EVT cells embed. Recently Bulmer et al. showed that during SpA remodeling, VSMC migrate outside of the artery, and this phenomenon is enhanced in the presence of EVT (Bulmer et al., 2012). While the mechanisms by which EVTs remodel SpA remain to be fully elucidated, available data are consistent with the hypothesis that EVT directly interact with VSMC of the uterine spiral arteries. We propose that the releases of nanoparticles (i.e., exosomes) that contain specific effector molecules (e.g., proteins and miRNAs) are released from EVT and affect the loss of VSMC.

Exosomes (30–100 nm) are nanovesicles released when late endosomes fuse with the cell membrane (Thery, 2011; Salomon et al., 2013a). Exosomes interact with target cells via multiple pathways including: by directly activating target cell membrane receptors; by modifying the extracellular *milieu* of the target cell; and by fusing with the cell membrane and releasing the molecular cargo into the target cell (Pegtel et al., 2010). Their molecular cargo is: cell-specific (Kobayashi et al., 2014); regulated by tissue physiology and cellular function; and fundamental to their bioactivity.

Exosomes are identified in cell-conditioned media and body fluids indicate that they can be released from different types of cells (Vlassov et al., 2012). Recently, the role of exosomes isolated from placental cells (Salomon et al., 2013a,b) and other cell types (Chen et al., 2014; Lee et al., 2014; Yoon et al., 2014) on cell migration has been established. Exosomes released from first trimester placental mesenchymal stem cells (pMSC) increase endothelial cell migration and vascular tube formation *in vitro* (Salomon et al., 2013a). Similarly, cytotrophoblast-derived exosomes increase EVT migration *in vitro* (Salomon et al., 2013b).

Consistent with the proposal that exosomal signaling regulates cell migration and invasion, proteins associated with actin cytoskeleton, growth hormone, and VEGF signaling have been identified within exosomes. The effect of EVT-derived exosomes on VSMC migration, however, remains to be established. We, therefore, hypothesize that exosomes released by EVT act paracellularly to promote VSMC migration and thus contributing to SpA remodeling. The aims of this study were: (1) to compare the exosome release and exosomal protein composition derived from EVT cell lines from different origin (JEG-3 and HTR-8/SVneo); and (2) to establish the effect of exosomes from both JEG-3 and HTR-8/SVneo cells on human VSMC migration.

Numerous human trophoblastic cell lines have been established, which basically originated from normal tissues or from pathological tissues. JEG3 is a choriocarcinoma cell line cloned from primary choriocarcinoma (Kohler and Bridson, 1971), and HTR8/SVneo is a transformed extravillous trophoblast cell line established by immortalizing primary EVT cells via transfection with simian virus 40 large T antigen (SV40) (Graham et al., 1993); both cell lines are frequently used as models of physiologically invasive extravillous trophoblast. EVT invasion into the myometrium is a critical process for remodeling the uterine spiral artery (in this stage EVT interact with VSMC), however, the invasiveness capacity between these two cells lines are different. HTR-8/SVneo have significantly higher invasion capacity than JEG-3 (Suman and Gupta, 2012). Moreover, differences between these two cells lines are not just in the invasion capacity, but also in their miRNA profiles (Morales-Prieto et al., 2012) as well as their protease (e.g., metalloproteases-9) expressions (Suman and Gupta, 2012), however, functional differences between exosome vesicles derived from JEG-3 and HTR-8/SVneo remain to be established. Previously, these cells lines have been validated and used routinely as models of EVT function (Suman and Gupta, 2012; Weber et al., 2013).

The aim of this study was to test the hypotheses that: (1) exosomes from EVT act paracellularly to promote VSMC migration; and (2) The release, protein content and bioactivity of exosomes is cell origin-dependent (i.e., EVT cell lines from choriocarcinoma and chorionic villi). The effects of exosomes isolated from the EVT-like cell lines, JEG-3 and HTR-8/SVneo cells on VSMC migration were assessed.

The data obtained are consistent with the hypothesis that the function of EVT-derived exosomes is cell origin specific and showed differences in the release, content and effects on VSMC migration. EVT may communicate with VSMC during SpA remodeling, stimulating their migration through these specific nanovesicles (i.e., exosomes).

## Materials and methods

### Materials

Medium RPMI 1640, Medium 231, Smooth Muscle Growth Supplement (SMGS), glutamine, antibiotics, HEPES, and phosphate buffered saline (PBS) were obtained from Life Technologies Corporation (Mulgrave, Victoria, Australia). CD63 ELISA kits were obtained from SBI (ExoELISA™, System Biosciences, Mountain View, CA).

### Cell culture

All experimental procedures were conducted within an ISO17025 accredited (National Association of Testing Authorities, Australia) research facility. All data were recorded within a 21 Code of Federal Regulation (CFR) part 11 compliant electronic laboratory notebook (Irisnote, Redwood City, CA, USA). JEG-3 human choriocarcinoma cell line was purchased from the European Collection of Cell Cultures (Porton Down, Salisbury, UK). The HTR-8/SVneo cell line was kindly donated by Dr. Charles H. Graham (Queen's University, Ontario, Canada). HTR-8/SVneo was established by the transfection of trophoblast cells isolated from first trimester villous explants, with a gene encoding simian virus 40 large T antigen to immortalize them (Graham et al., 1993). JEG-3 and HTR-8/SVneo cells were maintained in phenol red-free RPMI 1640 medium supplemented with 10% heat-inactivated fetal bovine serum, 1% non-essential amino acids, 1 mM sodium pyruvate and 100 U/mL penicillin, and 100 mg/mL streptomycin. Cultures were maintained at 37°C and humidified under an atmosphere of 5% CO_2_-balanced N_2_ to obtain 8% O_2_ (pO_2_ ~54 mmHg) in an automated PROOX 110-scaled hypoxia chamber (BioSphericsacona, NY, USA). Cells were subcultured with dissociation media, TrypLE™ Express (Life technologies, USA) and cellular viability was determined by Trypan Blue exclusion and Countess® Automated cell counter (Life Technologies, USA).

Human Vascular Smooth Muscle cells (hVSMC) were purchased from LONZA (Lonza Group Ltd.). VSMC were cultured in 231 media (Life Technologies Corporation) supplemented with SMGS, 100 U/ml penicillin, and 100 μg/ml streptomycin, at 37°C and humidified under an atmosphere of 5% CO_2_-balanced N_2_ to obtain 8% O_2_ (pO_2_ ~54 mmHg) in an automated PROOX 110-scaled hypoxia chamber (BioSpherics™, Lacona, NY, USA).

The *in vitro* cell migration rates of JEG-3 and HTR-8/SVneo we established using real-time cell imaging system (IncuCyte™ live-cell ESSEN BioScience Inc., Ann Arbor, MI, USA). Using a scratch assay format, cell were imaged every 3 h to monitor cell migration as previously described (Salomon et al., 2013b).

### Exosome isolation

Exosomes were isolated from cell-free JEG-3 and HTR-8/SVneo- conditioned media as previously described (Salomon et al., 2013a; Kobayashi et al., 2014). In brief, cell-conditioned media was centrifuged at 300 × g for 15 min, 2000 × g for 30 min, and 12,000 × g for 45 min to remove whole cells and debris. The resultant supernatant were passed through a 0.22 μm filter sterilize Steritop™ (Millipore, Billerica, MA, USA) and then centrifuged at 120,000 × g for 70 min (Thermo Fisher Scientific Ins., Asheville, NC, USA, Sorvall, SureSpin™ 630/36, fixed angle rotor). The pellet was resuspended in PBS, washed and re-centrifuged (120,000 × g, 75 min). The pellet was resuspended in PBS, layered on a cushion of 30% (w/v) sucrose and centrifuged at 110,000 g for 75 min. The fraction containing exosomes [~3.5 ml, 1.127 density using OPTi digital refractometer (Bellingham^+^Stanley Inc., Lawrenceville, GA, USA)] was recovered using a Pulse-Free Flow Peristaltic Pump with a flow rate range of 1 ml per min (GILSON Miniplus® model 3) and Fraction Collector (GILSON FC 203B model) and diluted in PBS, and then ultracentrifuged at 110,000 × g of 70 min. Recovered exosomes were resuspended in 50 μl PBS and their protein contents were determined using the Bradford assay (Bio-Rad DC). Exosome samples (5 μl) were prepared by adding RIPA buffer (50 mM Tris, 1% Triton × 100, 0.1% SDS, 0.5% DOC, 1 mM EDTA, 150 mM NaCl, protease inhibitor) directly to exosomes suspended in PBS and sonicated at 37°C for 15 s three times to disrupt exosomes and solubilise the proteins. Bovine serum albumin (BSA) diluted in RIPA buffer and PBS mixture (1:1) were prepared as protein standards (0, 200, 400, 600, 800, 1000, 1500 μg/mL). Standards and samples (exosomes) were transferred to 96-well plates and procedures outlined by the manufacture were followed. In brief, alkaline copper tartrate solution (Bio-Rab Laboratories, Hercules, CA, USA) and dilute Folin Reagent (Bio-Rab Laboratories,) were added to the samples and incubated for 15 min. The absorbance was read at 750 nm with Paradigm Detection Platform (Beckman Coulter, USA).

### Nanoparticle tracking analysis (NTA)

NTA measurements were performed using a NanoSight NS500 instrument (NanoSight NTA 2.3 Nanoparticle Tracking and Analysis Release Version Build 0033) following the manufacturer's instructions. The NanoSight NS500 instrument measured the rate of Brownian motion of nanoparticles and consists in a light scattering system that provides a reproducible platform for specific and general nanoparticle characterization (NanoSight Ltd., Amesbury, UK). Samples were processed in duplicate and diluted with PBS over a range of concentration to obtain between 10 and 100 particles per image (optimal ~50 particles × image) before the analysis with the NTA system. The samples were mixed before introducting into the chamber (temperature: 25°C and viscosity: 0.89 cP) and the camera level set to obtain an image that had sufficient contrast to clearly identify particles while minimizing background noise with video recording (camera level: 10 and capture duration: 60 s). Afterwards, the capture videos (2 videos per sample) were processed and analyzed. A combination of high shutter speed (450) and gain (250) followed by manual focusing enables optimum visualization of the maximum number of vesicles. A minimum of 200 completed tracks per video were collected in duplicate for each sample analyzed. NTA post acquisition settings were optimized and kept constant between samples (Frames Processed: 1496 of 1496, Frames per Second: 30, camera shutter: 20 ms; Calibration: 139 nm/pixel, Blur: 3 × 3; Detection Threshold: 10; Min Track Length: Auto; Min Expected Size: Auto), and each video was then analyzed to give the mean, mode, and median particle size together with an estimated number of particles. An Excel spreadsheet (Microsoft Corp., Redmond, Washington) was also automatically generated, recording the concentration at each particle size.

### Transmission electron microscopy

Exosome pellets (as described above, 30 μg protein) were fixed in 3% (w/v) glutaraldehyde and 2% paraformaldehyde in cacodylate buffer, pH 7.3. Exosome samples were then applied to a continuous carbon grid and negatively stained with 2% uranyl acetate. The samples were examined in an FEI Tecnai 12 transmission electron microscope (FEI™, Hillsboro, OR, USA).

### Quantification of cell-derived exosome

The concentration of exosomes from EVT cells lines (JEG-3 and HTR-8/SVneo) in maternal circulation was expressed as total immunoreactive exosomal CD63 (ExoELISA™, System Biosciences, Mountain View, CA). Briefly, 10 μg of exosomal protein was immobilized in micro-titer plate wells and incubated overnight (binding step). Plates were washed three times for 5 min using a wash buffer solution and then incubated with exosome specific primary antibody (CD63) at room temperature (RT) for 1 h on a shaker. Plates were washed and incubated with secondary antibody (1:5000) at RT 1 h on a shaker. Plates were washed and incubated with Super-sensitive TMB ELISA substrate at RT for 45 min while shaking. The reaction was terminated using Stop Buffer solution. Absorbance was measured at 450 nm. The number of exosomes/ml, (ExoELISA™ kit) was obtained using an exosomal CD63 standard curve calibrated against nanoparticle tracking data (i.e., number of exosomes, NanoSight™). The coefficients of intra- and inter-assay variations were less than 8%.

### Effect of exosomes on vascular smooth muscle cells (VSMC) migration

VSMC were cultured in 231 media supplemented with 0.2% FBS-exosome free in 96-well culture plate (Corning Life Science, Tewksbury, MA, USA) according to the manufacturer's instructions for 18–24 h. Firstly, we analyzed the effect of cell proliferation on cell migration assay in our experimental conditions using an anti-proliferative drug Mitomycin C (SIGMA-ALDRICH). Cells were plated (1 × 10^5^ cells per well) onto 24-well plate, and after 24 h Mitomycin C (50 and 100 ng/ml) was added to the cell for 48 h. Cell proliferation was quantified (time-curve) by measuring the cell confluence using a real time imagining system IncuCyte™. Simultaneously, VSMC migration assay in the presence of Mitomycin C (50 and 100 ng/ml) for 48 h was performed.

During experiments, VSMC cells were incubated in the presence (treatment: 100 μg exosomal protein/ml) or absence (control) of exosomes for up to 48 h under 8% O_2_ (*n* = 12). The concentration used in this study was based upon exosome dose-response curves from our previously published studies (Salomon et al., 2013a,b, 2014). Exosomes were subjected to heat inactivation (30 min at 65°C) or sonication for 30 min (sonicator bath) before the incubation on VSMC. Cell migration was assessed using a scratch assay format. A scratch was made on confluent monolayers using a 96-pin WoundMaker™ (BioScience Inc, Ann Arbor, MI, USA). Wound images were automatically acquired and registered by the IncuCyte™ software system. CellPlayer™ 96-Well Invasion Assay software was use to fully automate data collection. Data were processed and analyzed using IncuCyte™ 96-Well Cell Invasion Software Application Module. Data are presented as the Relative Wound Density (RWD, Eizen, v1.0 algorithm). RWD is a representation of the spatial cell density in the wound area relative to the spatial cell density outside of the wound area at every time point (time-curve). Migration assays were performed in the presence of Mitomycin C (100 ng/ml) to minimize any confounding effects of cell proliferation. The rate of wound closure was compared using the half-maximal stimulatory time (ST_50_) and area under the time course curve (AUC).

### Exosome internalization

For exosome uptake analysis, the exosomes pellet (before purification using 30% sucrose cushion) isolated from JEG-3 and HTR-8/SVneo cells was resuspended in PBS and stained with PKH67 green fluorescent cell linker kit follow the manufacturer's instructions (Sigma-Aldrich). The staining reaction was stopped after 5 min with exosome-free FBS. Exosomes were then purified using 30% sucrose cushion as described above. Exosomes-PKH67 (100 μg/ml from JEG-3 and HTR-8/svneo cells) were incubated on VSMC for 24 h and exosome uptake was quantified using IncuCyte™ (fluorescent mode). Simultaneously, VSMC were grown in glass bottom chamber slides, and labeled exosomes (100 μg/ml) were added to subconfluent cells. After 24 h, VSMC were washed and fixed using 2% paraformaldehyde for 5 min at RT. Cells were immediately analyzed using a Zeiss fluorescence microscope (Axio Imager M1, Zeiss EC Plan-NEOFLUAR 40×/0.75, the Carl Zeiss Group, Germany) with FITC and DAPI absorbance setting.

### Proteomic analysis of cytotrophoblast derived-exosomes by mass spectrometry (MS)

Isolated exosomes from JEG-3 and HTR-8/SVneo were solubilized in 8 M urea in 50 mM ammonium bicarbonate, pH 8.5, and reduced with DTT for 1 h. Proteins were then alkylated in 10 mM iodoacetic acid (IAA) for 1 h in the dark. The sample was diluted to 1:10 with 50 mM ammonium bicarbonate and digested with trypsin (20 μg) at 37°C for 18 h. The samples were desalted by solid phase extraction using a STAGE tip protocol (Stop and go extraction tips for matrix-assisted laser desorption/ionization, nano-electrospray, and LC/MS sample pre-treatment in proteomics). The eluted peptides were dried by centrifugal evaporation to remove acetonitrile and redissolved in Solvent A. The resulting peptide mixture was analyzed by liquid chromatography (LC)/mass spectrometry (MS) LC-MS/MS on a 5600 Triple TOF mass spectrometer (AB Sciex, Framingham, USA) equipped with an Eksigent Nanoflow binary gradient HPLC system and a nanospray III ion source. Solvent A was 0.1% formic acid in water and solvent B was 0.1% formic acid in acetonitrile. MS/MS spectra were collected using Information Dependent Acquisition (IDA) using a survey scan (m/z 350–1500) followed by 25 data-dependent product ion scans of the 25 most intense precursor ions. All mass spectra were analyzed using the Mascot and Protein Pilot search engines against the Swissprot-database with the species set as human. Positive identifications were ascribed where Mascot scores were greater than 30. False discovery rate (FDR) was estimated using a reversed sequence database. Finally, proteins identified were submitted to bioinformatic pathway analysis (Ingenuity Pathway Analysis [IPA]; Ingenuity Systems, Mountain View, CA; www.ingenuity.com).

### Statistical analysis

Data are represented as mean ± s.e.m. Comparisons between two group means were performed by unpaired Student's *t*-tests. Multiple groups were compared using the analysis of variance (ANOVA). *Post-hoc* analyses were used for pairwise comparisons (Bonferroni correction test). Statistical significance was defined as at least *p < 0.05*. Statistical analyses were preformed using commercially available packages (Stata 11, StatCorp, College Station, TX, USA and Prism 6, GraphPad Inc, La Jolla, CA 92037 USA).

## Results

### Characterization of EVT cell lines

**Figure 1:**
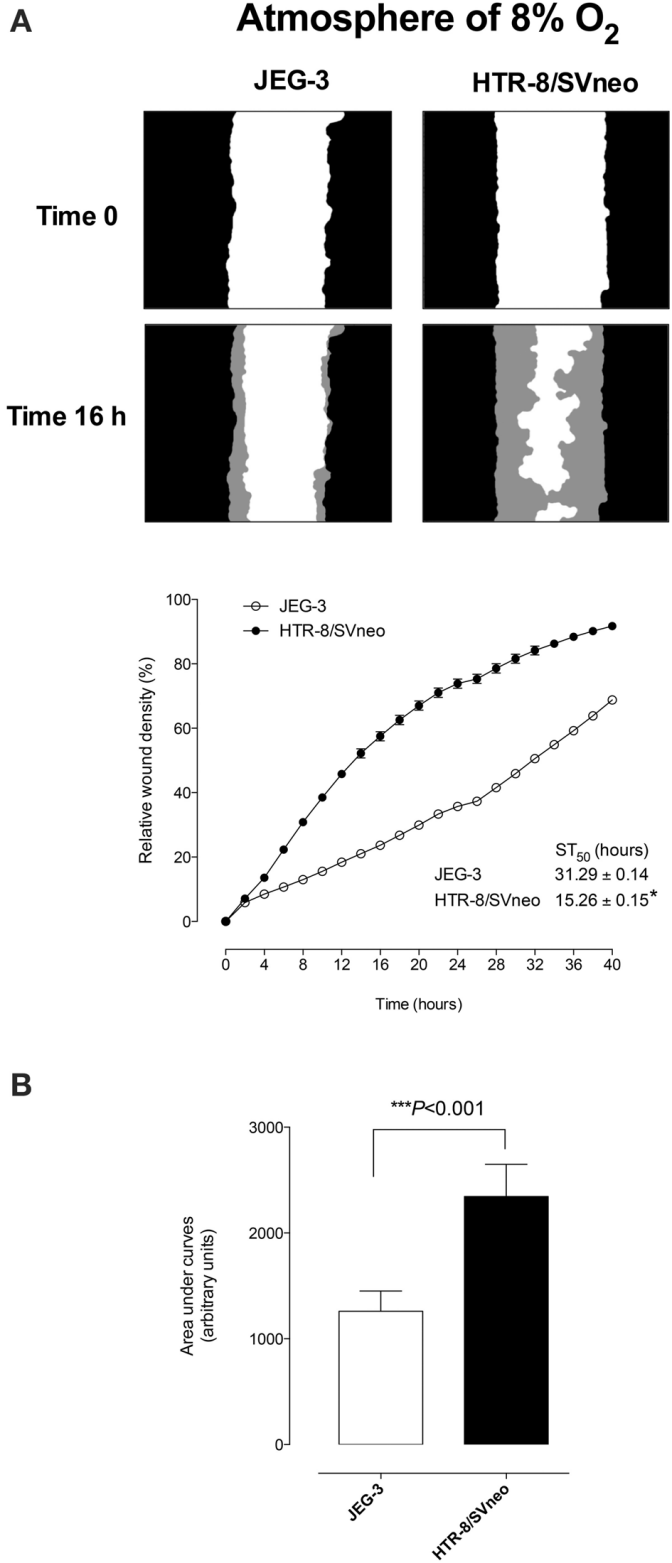
**Extravillous Trophoblast invasion**. JEG-3 and HTR-8/SVneo cells were grown to confluence in complete media. A wound was made using 96 well WoundMaker and cells were imaged with IncuCyte™ (Essen BioSciences, USA) every 4 h for 40 h (see Materials and Methods). **(A)** Top: Representative images of JEG-3 and HTR-8/SVneo cell migration at 16 h. The gray region denotes the area of the initial wound covered by advancing cells. Bottom: The time course of JEG-3 and HTR-8/SVneo migration. Insert: half-maximal stimulatory time (ST_50_). **(B)** Area under curves analysis from **(A)**. Data represented as mean ± s.e.m. (*n* = 12). In **(B)** ^***^p < 0.0001 vs. HTR-8/SVneo.

Figure 1A presents photomicrographs of wound closure over 40 h incubation for both cell lines and the percent change in relative wound density over time. The migration rate of HTR-8/SVneo was significantly greater than that observed for JEG-3 cells. Half-maximal stimulatory time (ST_50_) was 31.29 ± 0.14 h and 15.26 ± 0.15 h for JEG-3 and HTR-8SV/neo, respectively (*p* < 0.05, Figure 1A, bottom). Area under curves analysis showed that the migration of HTR-8SV/neo was ~2.0-fold greater than that obtained for JEG-3 cells (Figure 1B).

### Characterization of EVT cell line-derived exosomes

**Figure 2:**
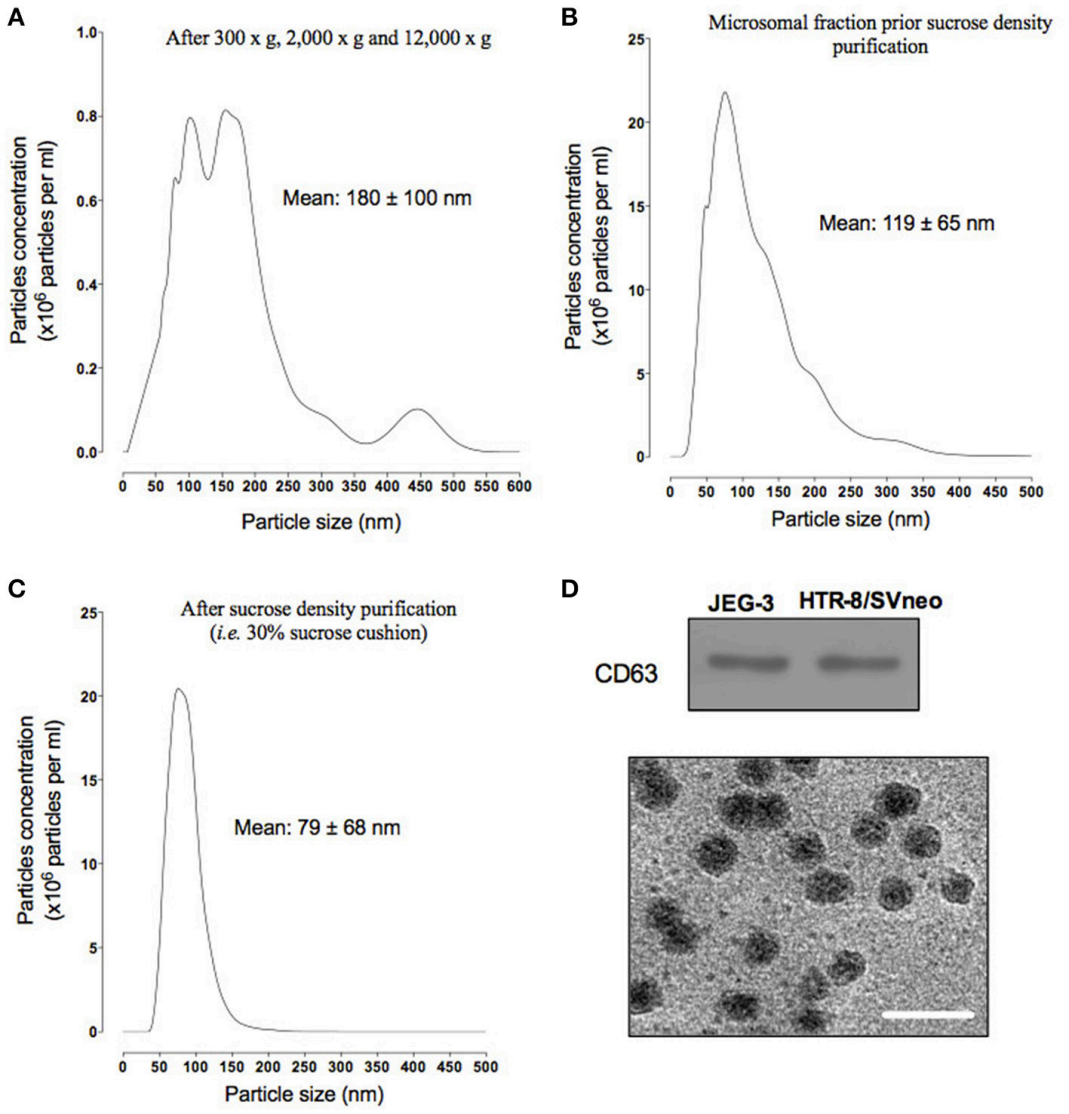
**Characterization of exosome from EVT cells**. Exosomes were isolated from JEG-3 and HTR-8/SV cells by differential and buoyant density centrifugation (see Materials and Methods). **(A–D)** shown representative particles size distribution of exosomes isolation steps from JEG-3 and HTR-8/SV cells. **(A)** After differential centrifugation (i.e., 300 × g, 2000 × g, and 12,000 × g). **(B)** Microsomal fraction prior sucrose density purification. **(C)** After sucrose density purification (30% sucrose cushion). **(D)** Top: representative Western blot for exosome markers: CD63. Bottom: representative electron micrograph exosome fractions, Scale bar 200 nm.

Defining vesicle size range is one of the standard metrics used to confirm that the vesicle preparation in not contaminated with non-exosome vesicles. After differential centrifugation (i.e., 300 × g, 2000 × g, and 12,000 × g), the supernatant was analyzed using a nanoparticles tracking analysis (NTA). Particle size distribution ranged from 30 to 500 nm, with an average of 180 ± 100 nm (Figure 2A). Supernatant was filtered and ultracentrifuged to obtain a particulate fraction (including exosomes particles) with a particle size ranged of 30–200 nm (with a mean diameter of 119 ± 65 nm, Figure 2B). The exosomes were purified by ultracentrifugation over a sucrose cushion and analyzed using a NTA. The exosome fractions displayed a particle size ranged from 30 to 150 nm in diameter (with mean of 79 ± 68 nm, Figure 2C). The presence of exosomal marker CD63 in exosomes isolated from both cell lines was confirmed by Western blot (Figure 2D, top). Finally, exosomes isolated from JEG-3 and HTR-8/SVneo were visualized by transmission electron microscopy (Figure 2D, bottom). Exosomes were identified as small vesicles between 40 and 100 nm. No significant differences in NTA data, CD63 expression and electron microscopy were identified between cell lines.

### Exosome release from EVT cell lines

**Figure 3:**
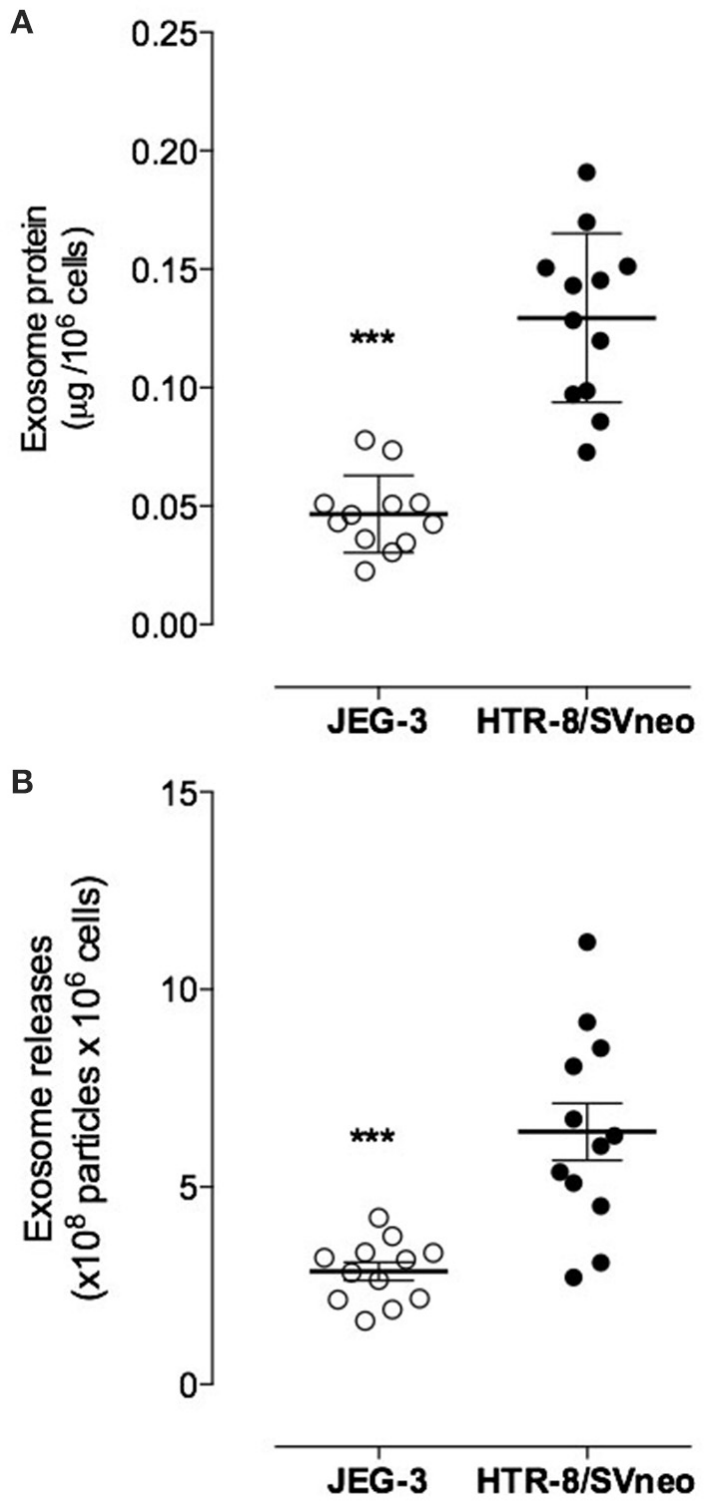
**Exosome releases from EVT cell lines**. Exosomal protein and number of exosomes were quantified cell conditioned media from JEG-3 and HTR-8/SVneo cells using a colorimetric assay and ELISA kit, respectively. **(A)** exosomes concentration presented as μg exosomal protein per 10^6^ cells and **(B)** Number of exosomes particles. Data are presented as scatter dot plot and values are mean ± s.e.m. In **(A,B)** ^***^p < 0.001 vs. HTR-8/SVneo.

Exosomal protein release was expressed as total exosomal protein (i.e., particulate material with a buoyant density of 1.127 g/ml) per million cells/24 h. JEG-3 cells released 46 ± 16 ng exosomal protein per million cells per 24 h (*n* = 4; i.e., 4 different isolations from ~200 × 10^6^ cells each) whereas HTR-8/SVneo cells released 120 ± 35 ng exosomal protein per million cells per 24 h (*n* = 4, Figure 3A). HTR-8/SVneo cells released significantly more exosomes (~2.6-folds) in 24 h compared to JEG-3 cells (*p* < 0.0001). These results were confirmed by quantifying immunoreactive exosomal CD63. The number of exosomes (NEP) released from HTR-8/SVneo cells (6.39 × 10^8^ ± 2.5 × 10^8^/24 h) was ~2.3-fold greater (*p* < 0.0001) than that observed for JEG-3 cells (2.86 × 10^8^ ± 0.78 × 10^8^/24 h, Figure 3B).

### Effect of EVT-derived exosomes on cell migration

**Figure 4:**
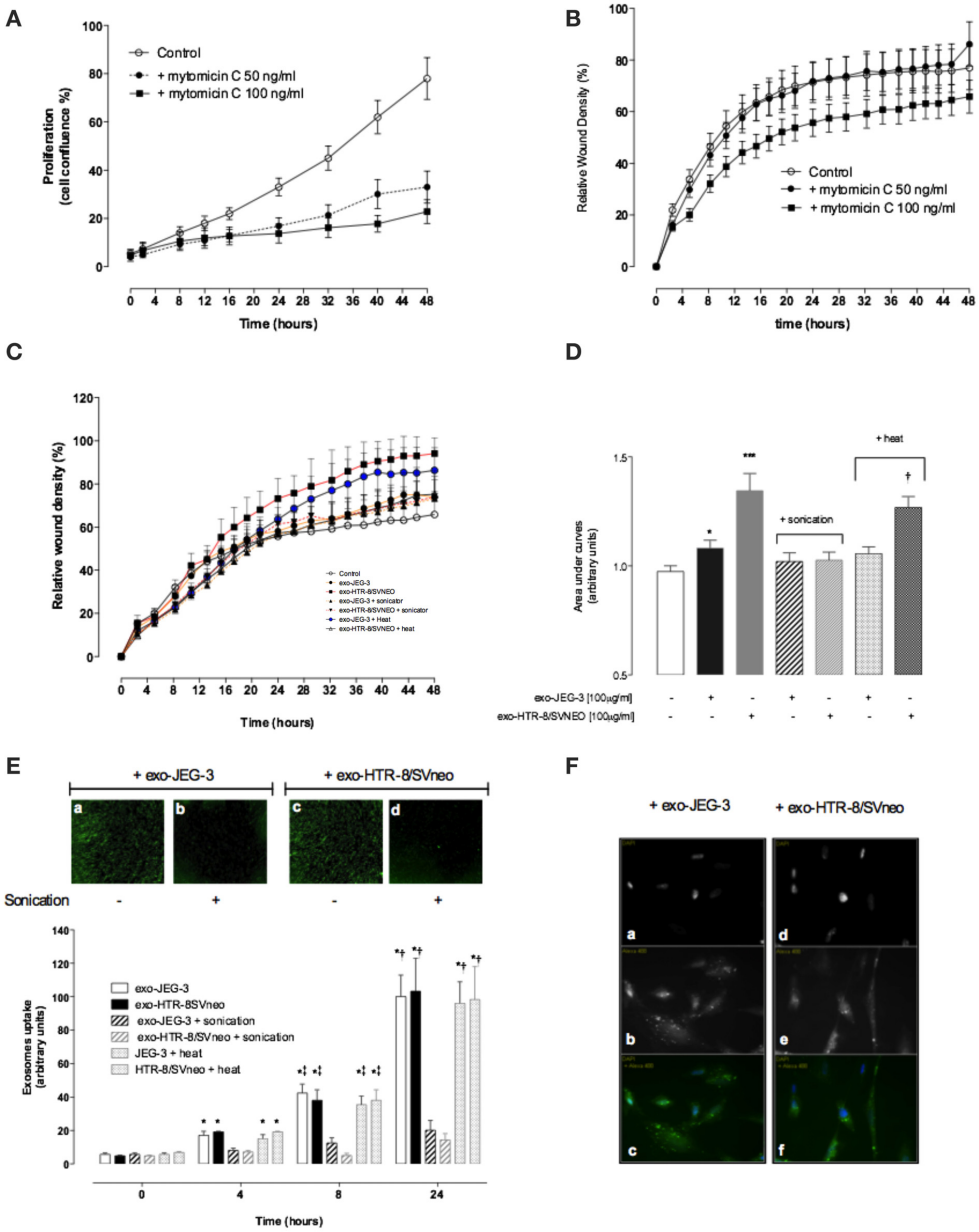
**EVT-derived exosomes effects on hVSMC migration**. hVSMC were grown to confluence in 231 media and a wound was made using 96 well WoundMaker (see Materials and Methods). hVSMC Migration was measured in absence or presence of 100 ug/ml of exosomes from JEG-3 and HTR-8/SVneo cells and mitomycin C (100 ng/ml) for 48 h. Exosome particles were subjected to sonication (+sonication) or heat inactivation (+heat) before exposure to hVSMC cells **(A,B)** hVSMC proliferation and migration in the presence of Mitomycin C, respectively. **(C)** Time course of wound closure for hVSMC expressed as relative wound density (%). **(D)** Area under curves from data in **(C)**. **(E)** Time-dependent uptake of exosomes using a real time imaging system analysis. Top: images after 24 h; Bottom: Graphical representation of exosome uptake. **(F)** Fluorescent microscopy analysis of exosome uptake (40X). Data represent an *n* = 12 well each point with 3 different cells culture. Values are mean ± SD. In **(D)** ^***^p < 0.001 vs. all condition except exo-HTR-8/SVneo + heat; and ^†^*p* < 0.05 vs. control. In **(E)** ^*^p < 0.001 vs. corresponding values at 0 h; ^‡^*p* < 0.05 vs. corresponding values control at 4 h; ^†^*p* < 0.05 vs. corresponding values control at 8 and 4 h.

A VSMC was used to establish the effects of EVT-derived exosomes on cell migration. An anti-proliferative drug Mitomycin C (50 and 100 ng/ml) reduces significantly (*p* < 0.01) VSMC proliferation (Figure 4A). A dose of 100 ng/ml decreased VSMC migration compared to 50 ng/ml of Mitomycin C and control (Figure 4B). The effects of exosomes (100 μg protein/ml) isolated from JEG-3 and HTR-8/SVneo cultured under 8% O_2_ on VSMC migration under 8% O_2_ are presented in Figures 4C,D. All experiments were done in the presence of Mitomycin C (100 ng/ml). The rate of wound closure was significantly increased in the presence of HTR-8/SVneo-derived exosome compared to control (-exosomes) as measured by ST_50_ (15.57 ± 0.32 vs. 25.09 ± 0.58, *p* < 0.01) (Table 1). Area under curves analysis showed that HTR-8/SVneo-derived exosome increased ~35 ± 0.2% VSMC migration compared to control. Similarly, exosomes from JEG-3 cells increased VSMC migration ~12 ± 0.1% compared to values in the absence of exosomes (control), however, the effect was smaller compared to exosomes from HTR-8/SVneo. Exosomes were exposed to heat inactivation before incubation on VSMC; however, heat inactivation did not affect the effect of exosomes on VSMC migration. In contrast, sonication completely abolished the HTR-8/SVneo and JEG-3-derived exosomes effect on VSMC migration.

**Table 1:** **Kinetic characteristic of exosome effects on hVSMC migration**.

**Condition**	**ST_50_ (hours)**
Control	25.09±0.58
+exo-JEG-3	21.83±0.49^‡^
+exo-HTR-8/SVneo	15.57±0.32^*^
+exo-JEG-3 + sonication	25.06±0.46
+exo-HTR-8/SVneo + sonication	23.27±0.48
+exo-JEG-3 + heat	24.14±0.35
+exo-HTR-8/SVneo + heat	19.43±0.34^†^

*The effect of exosomes isolated from JEG-3 and HTR-8/SVneo-conditioned media on hVSMC in vitro migration. Data are expressed as half-maximal Stimulatory Time (ST_50_ in hours) and represent the mean ± SD*.

* *p < 0.05 vs. control*;

† *p < 0.005 vs. control and +exo-HTR-8/SVneo*.

‡ *p < 0.005 vs. control*.

The internalization of exosomes labeled with PKH67 (green) in VSMC was quantified and visualized using The IncuCyte and a fluorescence microscope, respectively (Figures 4E,F). Exosome uptake by VSMC was observed in a time-dependent manner with the maximum at 24 h (Figure 4E, top panel a and c). Sonication abolished the uptake of fluorescent exosomes (Figure 4E, top panel b and d) compared to exosomes without sonication. Exosome uptake is presented as fluorescent per cell confluence normalized to maximum uptake of 100%. Heat inactivation did not affect the exosomes uptake by VSMC (Figure 4, lower panel). Finally, fluorescence microscope analysis showed an intracellular fluorescent in VSMC cells exposed to intact vesicles from JEG-3 and HTR-8/SVneo (Figure 4F).

### Proteomic analysis of EVT-derived exosome

**Figure 5:**
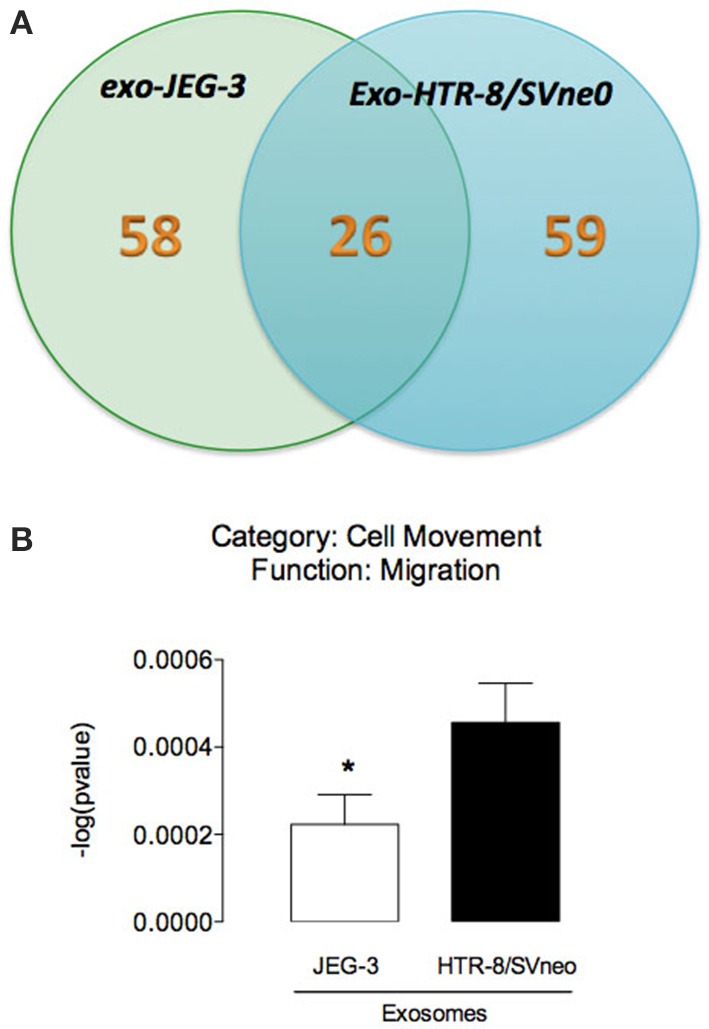
**Analysis of EVT cell-derived exosomes proteins. (A)** The Venn diagram represents the distribution of common and unique proteins identified by nanospray LC-MS/MS (ABSciex 5600) in exosomes released from JEG-3 and HTR-8/SVneo cells. **(B)** Comparison of biological function (cell movement and migration) identified by IPA core analysis. In **(B)** values are mean ± s.e.m. In **(B)** ^*^p < 0.05 vs. HTR-8/SVneo cells.

Mass spectrometry analysis identified over 140 exosomal proteins, including cell-line specific exosomal proteins (Table 2 and Figure 5A). Exosomal proteins isolated from JEG-3 and HTR-8/SVneo cells were associated with cellular movement and morphology, immune cell trafficking and cellular assembly and organization in accordance with Ingenuity Pathway Analysis (IPA) analysis. The canonical pathways associated with our exosomal proteins isolated from JEG-3 and HTR-8/SVneo cells and defined by IPA Core comparison analysis showed that the score (−log [*p*-value]) for proteins associated with cell movement and migration was significantly higher (~1.2-fold, *p* < 0.05) in HTR-8/SVneo-derived exosomes to compare to exosomes from JEG-3 cells (Figure 5B).

**Table 2:** **List of proteins identified in exosomes isolated from JEG-3 and HTR-8/SVneo cells**.

**Symbol**	**Entrez gene name**	**UniProt/Swiss-Prot accession**	**Location**	**Type(s)**
**COMMON PROTEINS IN EXOSOMES FROM JEG-3 and HTR-8/Svneo CELLS**				
A2M	Alpha-2-macroglobulin	A2MG_HUMAN	Extracellular space	Transporter
ACTB	Actin, beta	ACTB_HUMAN	Cytoplasm	Other
AHSG	Alpha-2-HS-glycoprotein	FETUA_HUMAN	Extracellular space	Other
ALB	Albumin	ALBU_HUMAN	Extracellular space	Transporter
APOE	Apolipoprotein E	APOE_HUMAN	Extracellular space	Transporter
APOM	Apolipoprotein M	APOM_HUMAN	Plasma membrane	Transporter
C3	Complement component 3	CO3_HUMAN	Extracellular space	Peptidase
EEF2	Eukaryotic translation elongation factor 2	EF2_HUMAN	Cytoplasm	Translation regulator
ENO1	Enolase 1, (alpha)	ENOA_HUMAN	Cytoplasm	Enzyme
F5	Coagulation factor V (proaccelerin, labile factor)	FA5_HUMAN	Plasma membrane	Enzyme
FGB	Fibrinogen beta chain	FIBB_HUMAN	Extracellular space	Other
FLNA	Filamin A, alpha	FLNA_HUMAN	Cytoplasm	Other
FN1	Fibronectin 1	FINC_HUMAN	Extracellular space	Enzyme
HSP90AA1	Heat shock protein 90 kDa alpha (cytosolic), class A member 1	HS90A_HUMAN	Cytoplasm	Enzyme
HSPA8	Heat shock 70 kDa protein 8	HSP7C_HUMAN	Cytoplasm	Enzyme
KRT1	Keratin 1	K2C1_HUMAN	Cytoplasm	Other
KRT10	Keratin 10	K1C10_HUMAN	Cytoplasm	Other
LDHB	Lactate dehydrogenase B	LDHB_HUMAN	Cytoplasm	Enzyme
MSN	Moesin	MOES_HUMAN	Plasma membrane	Other
PGK1	Phosphoglycerate kinase 1	PGK1_HUMAN	Cytoplasm	Kinase
PKM	Pyruvate kinase, muscle	KPYM_HUMAN	Cytoplasm	Kinase
PZP	Pregnancy-zone protein	PZP_HUMAN	Extracellular space	Other
THBS1	Thrombospondin 1	TSP1_HUMAN	Extracellular space	Other
VEPH1	Ventricular zone expressed PH domain-containing 1	MELT_HUMAN	Nucleus	Other
YWHAB	Tyrosine 3-monooxygenase/tryptophan 5-monooxygenase activation protein, beta	1433B_HUMAN	Cytoplasm	Transcription regulator
YWHAE	Tyrosine 3-monooxygenase/tryptophan 5-monooxygenase activation protein, epsilon	1433E_HUMAN	Cytoplasm	Other
**EXOSOMES FROM JEG-3 CELLS**				
ACTN1	Actinin, alpha 1	ACTN1_HUMAN	Cytoplasm	Other
ADAMTS13	ADAM metallopeptidase with thrombospondin type 1 motif, 13	ATS13_HUMAN	Extracellular space	Peptidase
AFP	Alpha-fetoprotein	FETA_HUMAN	Extracellular space	Transporter
AHCY	Adenosylhomocysteinase	SAHH_HUMAN	Cytoplasm	Enzyme
AMY2A	Amylase, alpha 2A (pancreatic)	AMYP_HUMAN	Extracellular space	Enzyme
APOA1	Apolipoprotein A-I	APOA1_HUMAN	Extracellular space	Transporter
ARGLU1	Arginine and glutamate rich 1	ARGL1_HUMAN	Other	Other
ATP10A	ATPase, class V, type 10A	AT10A_HUMAN	Plasma membrane	Transporter
C5	Complement component 5	CO5_HUMAN	Extracellular space	Cytokine
C7	Complement component 7	CO7_HUMAN	Extracellular space	Other
C4A/C4B	Complement component 4B (Chido blood group)	CO4A_HUMAN	Extracellular space	Other
C8B	Complement component 8, beta polypeptide	CO8B_HUMAN	Extracellular space	Other
CLEC11A	C-type lectin domain family 11, member A	CLC11_HUMAN	Extracellular space	Growth factor
COL6A1	Collagen, type VI, alpha 1	CO6A1_HUMAN	Extracellular space	Other
COLGALT1	Collagen beta(1-O)galactosyltransferase 1	GT251_HUMAN	Cytoplasm	Other
COMP	Cartilage oligomeric matrix protein	COMP_HUMAN	Extracellular space	Other
CPN1	Carboxypeptidase N, polypeptide 1	CBPN_HUMAN	Extracellular space	Peptidase
DGKQ	Diacylglycerol kinase, theta 110 kDa	DGKQ_HUMAN	Cytoplasm	Kinase
EEF1A2	Eukaryotic translation elongation factor 1 alpha 2	EF1A2_HUMAN	Cytoplasm	Translation regulator
F2	Coagulation factor II (thrombin)	THRB_HUMAN	Extracellular space	Peptidase
F10	Coagulation factor X	FA10_HUMAN	Extracellular space	Peptidase
F13A1	Coagulation factor XIII, A1 polypeptide	F13A_HUMAN	Extracellular space	Enzyme
FBLN1	Fibulin 1	FBLN1_HUMAN	Extracellular space	Other
FGG	Fibrinogen gamma chain	FIBG_HUMAN	Extracellular space	Other
GC	Group-specific component (vitamin D binding protein)	VTDB_HUMAN	Extracellular space	Transporter
GSN	Gelsolin	GELS_HUMAN	Extracellular space	Other
H2AFV	H2A histone family, member V	H2AV_HUMAN	Nucleus	Other
HBB	Hemoglobin, beta	HBB_HUMAN	Cytoplasm	Transporter
HIST1H1T	Histone cluster 1, H1t	H1T_HUMAN	Nucleus	Other
HIST1H2BB	Histone cluster 1, H2bb	H2B1B_HUMAN	Nucleus	Other
HSPA1L	Heat shock 70kDa protein 1-like	HS71L_HUMAN	Other	Other
IGLL1/IGLL5	Immunoglobulin lambda-like polypeptide 1	IGLL5_HUMAN	Plasma membrane	Other
ITIH1	Inter-alpha-trypsin inhibitor heavy chain 1	ITIH1_HUMAN	Extracellular space	Other
ITIH2	Inter-alpha-trypsin inhibitor heavy chain 2	ITIH2_HUMAN	Extracellular space	Other
ITIH3	Inter-alpha-trypsin inhibitor heavy chain 3	ITIH3_HUMAN	Extracellular space	Other
LINC00083	Long intergenic non-protein coding RNA 83	YA021_HUMAN	Other	Other
LRP1	Low density lipoprotein receptor-related protein 1	LRP1_HUMAN	Plasma membrane	Transmembrane receptor
LUM	Lumican	LUM_HUMAN	Extracellular space	Other
MYH9	Myosin, heavy chain 9, non-muscle	MYH9_HUMAN	Cytoplasm	Transporter
MYL6	Myosin, light chain 6, alkali, smooth muscle and non-muscle	MYL6_HUMAN	Cytoplasm	Other
NHSL2	NHS-like 2	NHSL2_HUMAN	Other	Other
OR2W3	Olfactory receptor, family 2, subfamily W, member 3	OR2W3_HUMAN	Plasma membrane	G-protein coupled receptor
PIF1	PIF1 5′-to-3′ DNA helicase	PIF1_HUMAN	Nucleus	Enzyme
PITRM1	Pitrilysin metallopeptidase 1	PREP_HUMAN	Cytoplasm	Peptidase
PLG	Plasminogen	PLMN_HUMAN	Extracellular space	Peptidase
PPP6R3	Protein phosphatase 6, regulatory subunit 3	PP6R3_HUMAN	Cytoplasm	Other
RAN	RAN, member RAS oncogene family	RAN_HUMAN	Nucleus	Enzyme
RDH13	Retinol dehydrogenase 13 (all-trans/9-cis)	RDH13_HUMAN	Cytoplasm	Enzyme
SERPINA7	Serpin peptidase inhibitor, clade A (alpha-1 antiproteinase, antitrypsin), member 7	THBG_HUMAN	Extracellular space	Transporter
SERPINF1	Serpin peptidase inhibitor, clade F (alpha-2 antiplasmin, pigment epithelium derived factor), member 1	PEDF_HUMAN	Extracellular space	Other
SERPINF2	Serpin peptidase inhibitor, clade F (alpha-2 antiplasmin, pigment epithelium derived factor), member 2	A2AP_HUMAN	Extracellular space	Other
THBS4	Thrombospondin 4	TSP4_HUMAN	Extracellular space	Other
TLN1	Talin 1	TLN1_HUMAN	Plasma membrane	Other
TUBA1B	Tubulin, alpha 1b	TBA1B_HUMAN	Cytoplasm	Other
TUBA4A	Tubulin, alpha 4a	TBA4A_HUMAN	Cytoplasm	Other
VTN	Vitronectin	VTNC_HUMAN	Extracellular space	Other
YWHAZ	Tyrosine 3-monooxygenase/tryptophan 5-monooxygenase activation protein, zeta	1433Z_HUMAN	Cytoplasm	Enzyme
ZNF480	Zinc finger protein 480	ZN480_HUMAN	Nucleus	Other
**EXOSOMES FROM HTR-8/SVneo CELLS**				
AASDH	Aminoadipate-semialdehyde dehydrogenase	ACSF4_HUMAN	Other	Enzyme
ACTN4	Actinin, alpha 4	ACTN4_HUMAN	Cytoplasm	Other
ALDOA	Aldolase A, fructose-bisphosphate	ALDOA_HUMAN	Cytoplasm	Enzyme
ANXA2	Annexin A2	ANXA2_HUMAN	Plasma membrane	Other
ANXA5	Annexin A5	ANXA5_HUMAN	Plasma membrane	Other
ANXA6	Annexin A6	ANXA6_HUMAN	Plasma membrane	Other
APOB	Apolipoprotein B	APOB_HUMAN	Extracellular space	Transporter
ARAP2	ArfGAP with RhoGAP domain, ankyrin repeat and PH domain 2	ARAP2_HUMAN	Cytoplasm	Other
ART4	ADP-ribosyltransferase 4 (Dombrock blood group)	NAR4_HUMAN	Nucleus	Enzyme
ATP1A1	ATPase, Na^+^/K^+^ transporting, alpha 1 polypeptide	AT1A1_HUMAN	Plasma membrane	Transporter
B2M	Beta-2-microglobulin	B2MG_HUMAN	Plasma membrane	Transmembrane receptor
BASP1	Brain abundant, membrane attached signal protein 1	BASP1_HUMAN	Nucleus	Transcription regulator
BMS1	BMS1 ribosome biogenesis factor	BMS1_HUMAN	Nucleus	Other
C9	Complement component 9	CO9_HUMAN	Extracellular space	Other
C7orf61	Chromosome 7 open reading frame 61	CG061_HUMAN	Other	Other
CD59	CD59 molecule, complement regulatory protein	CD59_HUMAN	Plasma membrane	Other
CHM	Choroideremia (Rab escort protein 1)	RAE1_HUMAN	Cytoplasm	Enzyme
CLTC	Clathrin, heavy chain (Hc)	CLH1_HUMAN	Plasma membrane	Other
COL12A1	Collagen, type XII, alpha 1	COCA1_HUMAN	Extracellular space	Other
FLNB	Filamin B, beta	FLNB_HUMAN	Cytoplasm	Other
FSCN1	Fascin homolog 1, actin-bundling protein (Strongylocentrotus purpuratus)	FSCN1_HUMAN	Cytoplasm	Other
GAPDH	Glyceraldehyde-3-phosphate dehydrogenase	G3P_HUMAN	Cytoplasm	Enzyme
GLP2R	Glucagon-like peptide 2 receptor	GLP2R_HUMAN	Plasma membrane	G-protein coupled receptor
GNAI2	Guanine nucleotide binding protein (G protein), alpha inhibiting activity polypeptide 2	GNAI2_HUMAN	Plasma membrane	Enzyme
GNB1	Guanine nucleotide binding protein (G protein), beta polypeptide 1	GBB1_HUMAN	Plasma membrane	Enzyme
GNG12	Guanine nucleotide binding protein (G protein), gamma 12	GBG12_HUMAN	Plasma membrane	Enzyme
HBD	Hemoglobin, delta	HBD_HUMAN	Other	Transporter
HIST1H1D	Histone cluster 1, H1d	H13_HUMAN	Nucleus	Other
HIST1H2BD	Histone cluster 1, H2bd	H2B1D_HUMAN	Nucleus	Other
HMCN1	Hemicentin 1	HMCN1_HUMAN	Extracellular space	Other
HSP90AB1	Heat shock protein 90 kDa alpha (cytosolic), class B member 1	HS90B_HUMAN	Cytoplasm	Enzyme
HSPB1	Heat shock 27 kDa protein 1	HSPB1_HUMAN	Cytoplasm	Other
HSPE1	Heat shock 10 kDa protein 1	CH10_HUMAN	Cytoplasm	Enzyme
ITGA3	integrin, alpha 3 (antigen CD49C, alpha 3 subunit of VLA-3 receptor)	ITA3_HUMAN	Plasma membrane	Other
ITGB1	Integrin, beta 1 (fibronectin receptor, beta polypeptide, antigen CD29 includes MDF2, MSK12)	ITB1_HUMAN	Plasma membrane	Transmembrane receptor
KRT9	Keratin 9	K1C9_HUMAN	Cytoplasm	Other
LGALS1	Lectin, galactoside-binding, soluble, 1	LEG1_HUMAN	Extracellular space	Other
MFGE8	Milk fat globule-EGF factor 8 protein	MFGM_HUMAN	Extracellular space	Other
MIF	Macrophage migration inhibitory factor (glycosylation-inhibiting factor)	MIF_HUMAN	Extracellular space	Cytokine
NCL	Nucleolin	NUCL_HUMAN	Nucleus	Other
NME2	NME/NM23 nucleoside diphosphate kinase 2	NDKB_HUMAN	Nucleus	Kinase
NRG1	Neuregulin 1	NRG1_HUMAN	Other	Growth factor
PARK7	Parkinson protein 7	PARK7_HUMAN	Nucleus	Enzyme
PGAM1	Phosphoglycerate mutase 1 (brain)	PGAM1_HUMAN	Cytoplasm	Phosphatase
PRDX1	Peroxiredoxin 1	PRDX1_HUMAN	Cytoplasm	Enzyme
PTGFRN	Prostaglandin F2 receptor inhibitor	FPRP_HUMAN	Plasma membrane	Other
RUVBL2	RuvB-like AAA ATPase 2	RUVB2_HUMAN	Nucleus	transcription regulator
S100A11	S100 calcium binding protein A11	S10AB_HUMAN	Cytoplasm	Other
SERPINA1	Serpin peptidase inhibitor, clade A (alpha-1 antiproteinase, antitrypsin), member 1	A1AT_HUMAN	Extracellular space	Other
SLC16A3	Solute carrier family 16 (monocarboxylate transporter), member 3	MOT4_HUMAN	Plasma membrane	Transporter
SLC2A1	Solute carrier family 2 (facilitated glucose transporter), member 1	GTR1_HUMAN	Plasma membrane	Transporter
STARD13	StAR-related lipid transfer (START) domain containing 13	STA13_HUMAN	Cytoplasm	Other
SYN1	Synapsin I	SYN1_HUMAN	Plasma membrane	Transporter
TMEM262	Transmembrane protein 262	YK025_HUMAN	Other	Other
TPI1	Triosephosphate isomerase 1	TPIS_HUMAN	Cytoplasm	Enzyme
TRRAP	Transformation/transcription domain-associated protein	TRRAP_HUMAN	Nucleus	Transcription regulator
TUBBP5	Tubulin, beta pseudogene 5	YI016_HUMAN	Other	Other
UBAC2	UBA domain containing 2	UBAC2_HUMAN	Cytoplasm	Enzyme
VIM	Vimentin	VIME_HUMAN	Cytoplasm	Other

*All mass spectra were analyzed using the Mascot and Protein Pilot search engines against the Swissprot-swissprot database with the species set as human (score over 30). Exosomal proteins identified by mass-spectrometry were analyzed with the Ingenuity Pathway Analysis (Ingenuity Systems, www.ingenuity.com). False discovery rate (FDR) was estimated using a reversed sequence database. List of total exosomal protein from cytotrophoblast cells exposed to different oxygen level are presented as Protein ID, Symbol, Entrez Gene Name, Location, and type*.

## Discussion

A tenet of contemporary obstetrics is that events that compromise placentation and the development of the materno-fetal exchange increase the risk of complications of pregnancy and contribute to poor pregnancy outcome. In particular, conditions that affect the differentiation and invasion of placental cells compromise placental perfusion and function (Jauniaux et al., 2006, 2010; Burton et al., 2009) and the subsequent growth and development of the fetus (Khong et al., 1987). EVT migrate into maternal decidua and myometrium and interact with VSMC in uterine spiral arteries. Conversion of these arteries is associated with the loss of VSMC from the vessel wall by migration out of the vessel. In this study we established that: (1) exosome isolated from EVT-like cell lines (JEG-3 and HTR-8/SVneo) with different cellular origins (choriocarcinoma and EVT) exhibit differences in their rate of release, protein content and bioactivity; and (2) both exosomes from JEG-3 and HTR-8/SVneo cells increase VSMC migration. However, the effect of exosomes from HTR-8/SVneo was significantly higher. Exosomes released from first trimester trophoblast cells (EVT), therefore, may regulate processes that play a key role in placentation and remodeling of the uterine spiral arteries.

The interactions between EVT and VSMC are not fully understood, in part due to difficulties of accessing first trimester samples and the lack of suitable animal models (Carter, 2007; Ackerman et al., 2013). We obtained nanovesicles with high purity. The data presented establish that the final preparation used display a size distribution and buoyant consistent with exosomes and the lack of significant contamination by microsomes. In this study, we quantified the release of exosomes from JEG-3 and HTR-8/SVneo cells (as indicated by immunoreactive exosomal CD63). The data obtained establish that exosome release is cell type specific and correlated with cell migration capacity. Consistent with these data, a correlation between exosome release and cell invasiveness has been described previously in ovarian cancer cells (Kobayashi et al., 2014).

The initial stages of uterine SpA remodeling involves a loss of the VSMC component by apoptosis, migration or a combination of both processes. In this study, we identify a novel exosomal signaling pathway by which EVT (HTR-8/SVneo cells) promote the migration of VSMC. Consistent with these data, Bulmer et al. (2012) observed that VSMC migration plays a major role in remodeling of the artery by migration into the decidua and vessel lumen and this phenomenon is enhanced in the presence of EVT cells (Bulmer et al., 2012). Based upon the available data, we propose that perivascular EVT release exosomes that interaction with VSMC and promote their migration out of SpAs and alter the vasoreactive of these vessels. This effect of exosomes on VSMC migration was cell-type specific (i.e., only exosomes from HTR-8/SVneo cells promote cell migration) and may reflect the different cellular origin of the two cell lines compared in this study. HTR-8/SVneo cells are derived by transfection of first trimester trophoblast cells (Graham et al., 1993). JEG-3 cells are derived from a choriocarcinoma (Kohler and Bridson, 1971). Difference in the molecular cargo carried by exosomes released from HTR-8/SVneo and JEG-3 cell may account for their different effects on target cell migration. Consistent with this suggestion, proteins involved in cell migration were differentially represented in exosomes isolated from the two-cell lines.

The exosomal content is highly dependent on the cell origin and on pre-conditioning of the cell. Exosomes function as a carrier of specific molecules such as, proteins, lipids, mRNA, and miRNA and can interact with neighboring cells or travel long distances in the bloodstream to reprogram the phenotype and regulate their function (Denzer et al., 2000). In this study, we identified unique proteins (58 and 59 proteins in exosome from JEG-3 and HTR-8/SVneo cells) and common proteins (26) between these EVT cell lines. Ingenuity Pathway Analysis (IPA) of exosomal proteins identified cell-dependent changes in cell movement and migration signaling pathways. Exosomes have been reported to express a diverse range of cell surface receptors, proteins (including, heat shock proteins, cytoskeletal proteins, adhesion molecules, membrane transport, and fusion proteins), mRNA and miRNA with the potential to affect the acute and long-term function of the cells with which they interact (Ambros, 2004). Recent data demonstrate that trophoblast-derived exosomes induce proinflammatory cytokines such IL-1 β in human macrophages cells (Atay et al., 2011). Furthermore, *in vitro* exposure of PBMC and dendritic cells to exosomal proteins induce differentiation of stem cells; suppression of activation of natural killer cells and macrophages; and stimulation of cell migration (Mincheva-Nilsson et al., 2006; Knight, 2008; Soo et al., 2012). Interestingly, protein analysis revealed that exosome release from cytrophoblast cells increases with low oxygen tension and their exosome promotes cell migration in extravillous cytotrophoblast (HTR-8/SVneo) (Salomon et al., 2013b).

The observed effects of HTR-8/SVneo-derived exosomes on VSCM migration were also dependent upon exosome structural integrity. Disruption of exosomes by sonication (Delorme-Axford et al., 2013) completely abolished their effect on VSMC migration. While the precise mechanisms by which sonication abolishes the effects of exosomes remains to be established, preventing exosomal fusion with VSMC cell membrane and the intracellular delivery of signaling molecules and/or the loss of capacity to appropriately present exosomal surface moieties to target cell receptors represent possible pathways and warrant further investigation. We establish in this study that exosome integrity is critical to mediating their effects on VSMC migration. It is not possible from the data obtained in this study to differentiate between sonication–induced disruption of exosome: uptake; fusion; activation of VSMC cell surface receptors, or a combination of these (and other) mechanisms. The elucidation of contribution of these mechanisms will require additional extensive studies to establish.

In conclusion, using *in vitro* EVT-like cell lines, we have demonstrated difference in release, composition and bioactivity between exosomes from JEG-3 and HTR-8/SVneo cells. Exosomes released from EVT may play a role in remodeling SpA by promoting migration of VSMC. Exosomal-induced VSCM migration is associated with increased representation of proteins involved in cell migration processes within their molecular cargo and dependent upon exosomal structural integrity. The identification of this EVT-VSMC exosomal communication pathway not only affords opportunity for developing biomarkers of placentation but also the assessment of exosome targeted therapeutic innervation strategies.

## Author contributions

Carlos Salomon, Sarah Yee, Katherin Scholz-Romero, Kanchan Vaswani, Miharu Kobayashi and David Kvaskoff contributed in generating experimental data. Carlos Salomon, Sebastian E. Illanes, Murray D. Mitchell and Gregory E. Rice contributed in discussion and reviewed/edited manuscript. Carlos Salomon and Gregory E. Rice wrote the manuscript and drew the figures.

### Conflict of interest statement

The authors declare that the research was conducted in the absence of any commercial or financial relationships that could be construed as a potential conflict of interest.
